# MicroRNAs associated with AGL6 and IAA9 function in tomato fruit set

**DOI:** 10.1186/s13104-023-06510-z

**Published:** 2023-09-30

**Authors:** Barbara Molesini, Federica Pennisi, Nicola Vitulo, Tiziana Pandolfini

**Affiliations:** Department of Biotechnology, University of Verona, Verona, 37134 Italy

**Keywords:** MicroRNA, RNA-Seq, Fruit set, Tomato, RNA silencing, Parthenocarpy

## Abstract

**Objective:**

Fruit set is triggered after ovule fertilization, as a consequence of the downregulation of ovary growth repressors, such as the tomato transcription factors Auxin/indole-3-acetic acid 9 (IAA9) and Agamous-like 6 (AGL6). In a recent work, we developed a method to silence *IAA9* and *AGL6* in tomato ovaries using exogenous dsRNAs. We also produced small RNA libraries from *IAA9*- and *AGL6*-silenced ovaries to confirm the presence of siRNAs, derived from exogenous dsRNA, targeting *IAA9* and *AGL6*. The objective of this work is to exploit these sRNA libraries to identify miRNAs differentially expressed in *IAA9*- and *AGL6*-silenced ovaries as compared with unpollinated control ovaries.

**Results:**

We identified by RNA sequencing 125 and 104 known and 509 and 516 novel miRNAs from reads mapped to mature or hairpin sequences, respectively. Of the known miRNAs, 7 and 45 were differentially expressed in *IAA9*- and *AGL6*-silenced ovaries compared to control ones, respectively. Six miRNAs were common to both datasets, suggesting their importance in the fruit set process. The expression pattern of two of these (miR393 and miR482e-5p) was verified by stem-loop qRT-PCR. The identified miRNAs represent a pool of regulatory sRNAs potentially involved in tomato fruit initiation.

**Supplementary Information:**

The online version contains supplementary material available at 10.1186/s13104-023-06510-z.

## Introduction

MicroRNAs (miRNAs) are small non-coding RNAs of ~ 20–24 nt involved in the regulation of target mRNAs. Most miRNAs are transcribed from miRNA genomic loci by RNA polymerase II that generates a precursor, called primary miRNA (pri-miRNA), characterised by a stem-loop structure. In plants, miRNA maturation occurs in the nucleus, and for the accurate processing of the pri-miRNA, the action of a multiprotein complex composed by the RNase type III Dicer-like 1 (DCL1) enzyme, Hyponastic Leaves 1 (HYL1) double-stranded RNA-binding protein, and zinc finger Serrate (SE) protein, is required [1–4]. DCL1 cleaves the pri-miRNA producing a miRNA precursor (pre-miRNA), and the processing continues until the mature miRNA/miRNA* duplex is released [5]. The duplex, characterised by 2-nt overhangs, as well as a 5′ phosphate (P) and a 3′ OH [6], is exported into the cytoplasm by HASTY, the orthologue of mammalian EXPORTIN5 [7]. The miRNA/miRNA* duplex undergoes 2′-O-methylation by the S-adenosyl methionine (SAM)-dependent methyltransferase HEN1 at the 3′ end. The miRNA is selectively loaded into ARGONAUTE 1 (AGO1) to form an active miRNA-induced silencing complex, which negatively regulates target mRNA expression at the post-transcriptional level, mainly through mRNA cleavage and translational repression [8]. Several evidence shows that functional mature miRNAs can originate from the 5′ side of the pre-miRNA (designed as the ‘5p’ strand) or from the 3’ side (also referred to as the ‘3p’ strand), pairing on different targets [9]. In plants, miRNAs and their targets are evolutionarily conserved between different plant species. The targets of miRNAs generally encode transcription factors (TFs) and regulatory proteins, disease resistance genes, metabolic enzymes, and transporters [10]. Functional characterisation of many miRNAs and their targets in different crop species, including *Solanum lycopersicum*, has revealed multiple roles in several biological processes such as vegetative and reproductive development, responses to phytohormones, abiotic and biotic stresses, and fruit development [10].

Most miRNAs are implicated in complex regulatory networks, including that underlying fruit set, a very early phase of fruit development that marks the transition from a quiescent ovary to the growing fruit after successful fertilization of the flower. For instance, tomato plants overexpressing miR167, which targets Auxin Response Factors 6/8 (ARF6/8), are unable to generate fruits [11]. Parthenocarpy, which is the fruit set in the absence of fertilization, was achieved by overexpressing miR159, whose targets, the transcription factors GAMYB1 and GAMYB2, are regulated by gibberellin [12]. Knockdown by miR166 of *Sl*HB15A, belonging to class III homeodomain leucine-zipper transcription factors, caused aberrant ovule formation correlating with parthenocarpic fruit set under adverse temperature conditions [13]. In the same work, *Sl*HB15A was shown to be a key regulator of hormonal activity, repressing auxin and activating ethylene biosynthesis and signalling within ovules [13], corroborating the crucial role played by hormones, in concert with TFs, in regulating fruit set [14, 15].

One possible approach to study the implication of miRNAs in fruit set is to dissect the ovary growth from ovule fertilization by inhibiting the activity of ovary growth repressors prior to flower anthesis. A key role in the repression of ovary growth before fertilization is played by two regulatory proteins: Auxin/indole-3-acetic acid 9 Transcription Factor (IAA9), and AGAMOUS-like6 (AGL6), a MADS-box MIKCC type II transcription factor. Downregulation of *IAA9* and *AGL6* in tomato induced parthenocarpic fruit development [16, 17]. In a previous study, we developed a transient silencing method to investigate the molecular changes occurring at fruit setting by downregulating *IAA9* and *AGL6*, using exogenous application of double-stranded RNA (dsRNA) coupled to layered double hydroxide (LDHs) to very young tomato flower buds [18]. The dsRNA delivery strategy caused the reduced expression of the target transcripts. We also produced small RNA-Seq libraries of *IAA9*- and *AGL6*-silenced ovaries and showed that the downregulation of the target genes was due to the formation of small interfering RNAs (siRNAs) derived from dsRNA processing. The same small RNA-Seq database is used in the present study to reveal alterations in the expression of known and novel miRNAs in *AGL6*- and *IAA9*-silenced ovaries. This work represents an extension of our previous research article, contributing to highlight the molecular changes underlying the fruit set process.

## Results

To identify miRNAs potentially involved in fruit set, we analyzed unpollinated tomato ovaries collected before anthesis, in which the expression of *IAA9* and *AGL6* was downregulated as a consequence of treatment with exogenous dsRNA [18]. Five days after the application of specific dsRNA, the transcript level of *IAA9* and *AGL6* was reduced by approximately 40 and 60%, respectively. These ovaries were used for the construction and sequencing of two sRNA libraries: *IAA9*- and *AGL6*-silenced (hereafter referred to as IAA9sil and AGL6sil). A third sRNA library was obtained from emasculated control (mock-treated) ovaries (ctrl). In each sample, the number of raw reads ranged from 26 to 30 millions (Table 1). Data cleaning was performed on the FASTQ file of the raw reads to obtain the final clean reads, which represent averagely 97% of the raw ones. Low quality reads, such as those with N > 10% (N means indeterminable), 5’ adapter contamination, sequences without a 3’ adapter or insert, with poly(A)/(T)/(G)/(C) tails, were discarded (Table 1). Unique clean reads between 18 and 30 nt were 2,864,283 (IAA9sil) 3,552,836 (AGL6sil) and 2,499,669 (ctrl) (Table 1).

**Table 1:** Quality control and data filtering

Sample	Total reads	N%>10%	5’ adapter contamination	3’ adapter null or insert null	With polyA/T/G/C	Clean reads	Clean reads (18–30 nt)
**ctrl**	30,618,662 (100%)	1836 (0.01%)	16,153 (0.05%)	684,938 (2.24%)	63,506 (0.21%)	29,852,229 (97.50%)	2,499,669
**AGL6sil**	26,439,854 (100%)	651 (0.00%)	33,216 (0.13%)	748,029 (2.83%)	75,428 (0.29%)	25,582,530 (96.76%)	3,552,836
**IAA9sil**	27,947,482 (100%)	1701 (0.01%)	28,256 (0.10%)	515,332 (1.84%)	59,739 (0.21%)	27,342,454 (97.84%)	2,864,283

Small RNA reads were initially mapped to the reference genome using Bowtie and then aligned to the plant miRNAs sequences deposited in the miRBase21 database. Globally, 125 and 104 known miRNAs were identified from reads mapping either to mature or hairpin sequences, respectively (Table 2).

**Table 2:** Summary of known and novel miRNAs in the samples

	Known miRNAs				Novel miRNAs			
Sample	Total	Ctrl	AGL6sil	IAA9sil	Total	Ctrl	AGL6sil	IAA9sil
**Mapped mature**	125	121	118	120	509	481	498	498
**Mapped hairpin**	104	103	104	102	516	495	508	506
**Mapped unique sRNA**	9387	3036	2999	3352	10,320	2998	4008	3314
**Mapped total sRNA**	3,595,509	1,064,907	1,453,322	1,077,280	144,286	35,050	70,909	38,327

Of these, reads mapping to mature sequences were 118, 120, and 121 in AGL6sil, IAA9sil, and ctrl, respectively (Table 2). The number of sRNAs aligned to miRNA hairpin sequences was 104, 102, and 103 in AGL6sil, IAA9sil, and ctrl, respectively (Table 2). The number of unique mapped sRNAs was similar in the three samples, whereas more total sRNA reads were detected in AGL6sil. We also analyzed the nucleotide bias at the first position for known miRNAs (Additional file 1). In all groups, miRNAs between 18 and 23 nt showed a preference for ”U”, whereas in miRNAs of 24–26 nt the preferred first base was ”A” (Additional file 1). In accordance with previous studies, miRNAs showed a prevalent length of 21 nt with ”U” at 5’-end (Additional file 1), features typical of the most plant miRNAs [19, 20].

Identification of novel miRNAs was performed using miREvo [21] and mirdeep2 [22] to predict miRNA by exploring the hairpin secondary structure of the precursors (Table 2). A total of 509 and 516 novel miRNAs were identified by annotation on the mature or hairpin sequence, respectively. The number of total and unique mapped sRNA was higher for AGL6sil than for the IAA9sil and ctrl. The first-base frequency of the new miRNAs of different lengths was also analysed, which revealed that in the three libraries, miRNAs of 18–22 nt preferentially started with '“U”, except for those of 20 nt which showed a preference for “G”. The highest percentage of miRNAs of 23–26 nt had “A” as the first base (Additional file 2). In the 3 libraries, the most abundant novel miRNAs were 24 nt in length with “A” as the most common base at 5’-end. In *Arabidopsis* and rice this long class of miRNAs has been shown to function as heterochromatic siRNAs to deposit repressive chromatin marks [20].

To find out differentially expressed miRNA in the* IAA9*- and *AGL6*-silenced ovaries, we normalized miRNA read count of the three libraries based on median values and then the fold change IAA9 vs. crtl and AGL6 vs. crtl was calculated and expressed as log_2_. The data were filtered considering a threshold of 50 for the total abundance of reads in the three libraries and fold changes ≥ 1.5 and ≤ − 1.5 (Table 3, Additional files 3 and 4). Seven (1 upregulated and 6 downregulated) and 45 (4 upregulated and 41 downregulated) known miRNAs were differentially expressed in IAA9sil and AGL6sil, respectively. Among these, 6 were differentially expressed in both libraries (i.e. sly-miR482e-5p; sly-miR167a, sly-miR530, sly-miR171b-5p, sly-miR394-3p, sly-miR393); five of these were downregulated in both libraries, and one showed an opposite pattern. The locus ID of the mRNA targets predicted for known miRNAs are reported in Additional file 3. Eighteen (7 upregulated and 11 downregulated) and 33 (17 upregulated and 16 downregulated) differentially expressed novel miRNAs were detected in the *IAA9*- and *AGL6*-silenced ovaries, respectively (Additional file 4). Four (novel_25; novel_170; novel_225; novel_522) were in common in both libraries with the same pattern of expression.

**Table 3:** Known differentially expressed miRNAs

miRNAs	Fold change (IAA9sil vs. ctrl)	Mature sequence	Number of target transcripts
sly-miR482e-5p	-1.75	UGUGGGUGGGGUGGAAAGAUU	10
sly-miR167a	-1.77	UGAAGCUGCCAGCAUGAUCUA	5
sly-miR395a	-1.76	CUGAAGUGUUUGGGGGAACUCC	4
sly-miR530	-3.04	AGGUGUAGGUGUUCAUGCAGA	3
sly-miR171b-5p	1.66	AUAUUGGUGCGGUUCAAUUAG	3
sly-miR394-3p	-1.68	AGGUGGGCAUACUGUCAACA	2
sly-miR393	-3.46	AUCAUGCGAUCUCUUCGGAAU	
**miRNAs**	**Fold change (AGL6sil vs. ctrl)**	**Mature sequence**	**Number of target transcripts**
sly-miR6024	-1.88	UUUUAGCAAGAGUUGUUUUACC	40
sly-miR172a	-4.70	AGAAUCUUGAUGAUGCUGCAU	18
sly-miR9476-5p	-3.56	UCUAGUCCUGCAUCUUUUUUU	16
sly-miR172c	-2.13	AGAAUCUUGAUGAUGCUGCAG	15
sly-miR164a-5p	-2.71	UGGAGAAGCAGGGCACGUGCA	11
sly-miR482e-5p	-4.01	UGUGGGUGGGGUGGAAAGAUU	9
sly-miR390a-5p	-3.27	AAGCUCAGGAGGGAUAGCACC	9
sly-miR482a	1.79	UUUCCAAUUCCACCCAUUCCUA	8
sly-miR10537	-2.12	AUUUACCCCAAGUUCGUUGUC	8
sly-miR9474-5p	1.90	UGUAGAAGUCAUGAAUAAAAUG	7
sly-miR171b-3p	-2.96	UUGAGCCGUGCCAAUAUCACG	6
sly-miR396a-3p	-4.63	GUUCAAUAAAGCUGUGGGAAG	5
sly-miR9472-5p	-4.71	UUUCAGUAGACGUUGUGAAUA	5
sly-miR167a	-2.75	UGAAGCUGCCAGCAUGAUCUA	5
sly-miR394-5p	-1.86	UUGGCAUUCUGUCCACCUCC	5
sly-miR5300	-1.96	UCCCCAGUCCAGGCAUUCCAAC	5
sly-miR169e-3p	-3.48	UGGCAAGCAUCUUUGGCGACU	5
sly-miR168a-5p	-1.76	UCGCUUGGUGCAGGUCGGGAC	4
sly-miR171a	-1.57	UGAUUGAGCCGUGCCAAUAUC	4
sly-miR9470-5p	-2.48	UGAAAUCCAUGAGCCUAAACU	4
sly-miR482d-5p	-2.58	GGAGUGGGUGGGAUGGAAAAA	4
sly-miR9475-3p	-1.53	CUACAAUGUAGAGAUCGUUUU	3
sly-miR171f	-3.08	UAUUGGCCUGGUUCACUCAGA	3
sly-miR164b-3p	-2.03	CACGUGUUCUCCUUCUCCAAC	3
sly-miR9476-3p	-1.85	AAAAAGAUGCAGGACUAGACC	3
sly-miR403-3p	-1.70	CUAGAUUCACGCACAAGCUCG	3
sly-miR530	-3.54	AGGUGUAGGUGUUCAUGCAGA	3
sly-miR171b-5p	-2.12	AUAUUGGUGCGGUUCAAUUAG	3
sly-miR168a-3p	-3.89	CCUGCCUUGCAUCAACUGAAU	2
sly-miR164a-3p	-5.53	CAUGUGCCUGUUUUCCCCAUC	2
sly-miR403-5p	-4.42	CGUUUGUGCGUGAAUCUAACA	2
sly-miR6027-5p	-2.20	AUGGGUAGCACAAGGAUUAAUG	2
sly-miR394-3p	-8.78	AGGUGGGCAUACUGUCAACA	2
sly-miR166c-5p	-4.62	GGGAUGUUGUCUGGCUCGACA	1
sly-miR171c	-1.99	UAUUGGUGCGGUUCAAUGAGA	1
sly-miR5304	-1.51	UCAAUGCUACAUACUCAUCCC	1
sly-miR397-3p	1.86	UCAACGCUAAACUCGAUCAUG	1
sly-miR9475-5p	-1.58	AACGAUCUCUACAUUGUAGGC	
sly-miR398a	3.20	UAUGUUCUCAGGUCGCCCCUG	
sly-miR390a-3p	-1.71	CGCUAUCCAUCCUGAGUUUUA	
sly-miR1919a	-2.24	ACGAGAGUCAUCUGUGACAGG	
sly-miR167b-5p	-2.34	UAAAGCUGCCAGCAUGAUCUGG	
sly-miR9471a-5p	-1.53	CAGGUGCUCACUCAGCUAAUA	
sly-miR9471b-5p	-3.37	GAGGUGCUCACUCAGCUAAUA	
sly-miR393	-1.87	AUCAUGCGAUCUCUUCGGAAU	

**Fig. 1:**
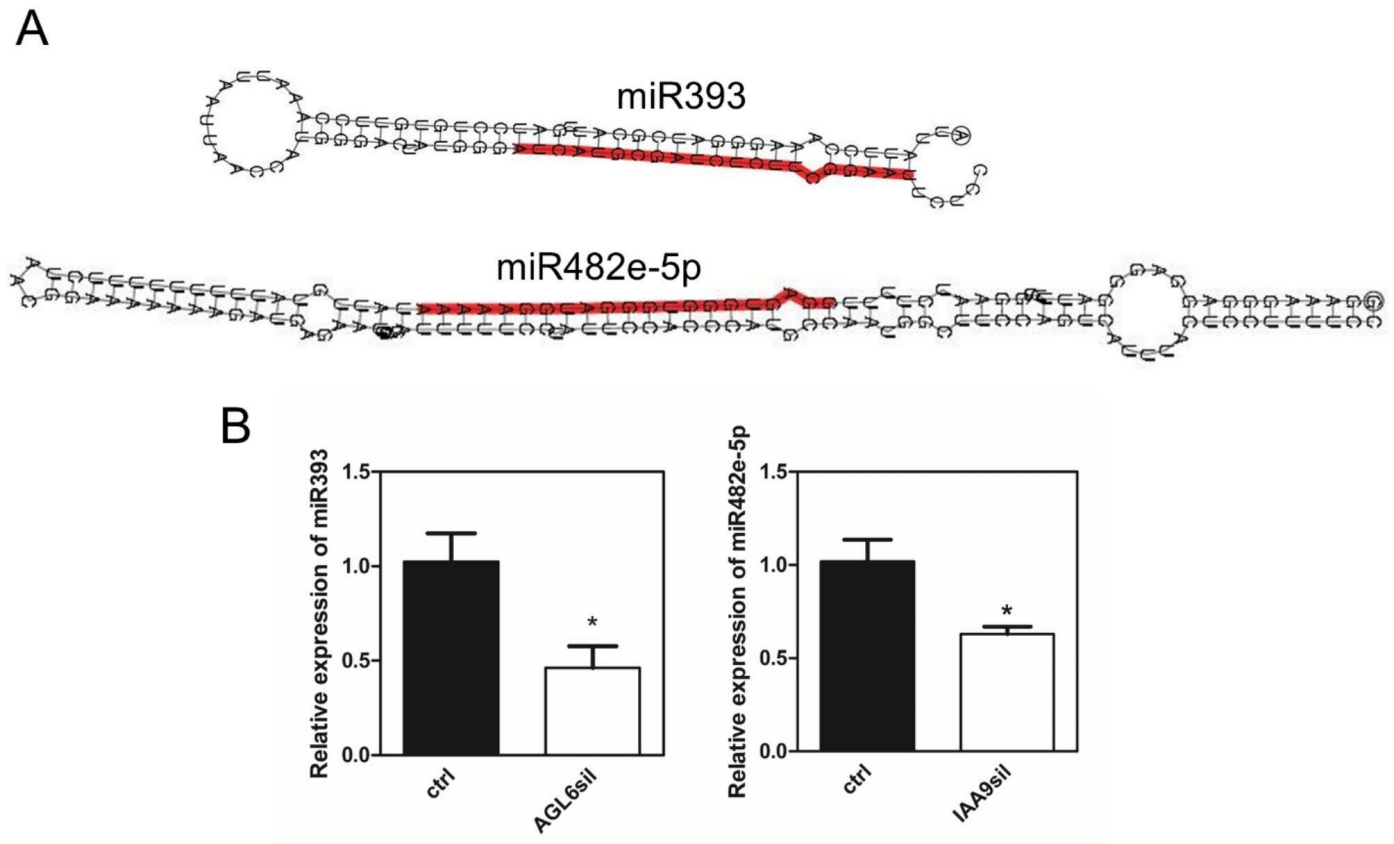
**A**, Secondary structure for miR393 and miR482e-5p. **B**, Expression of miR393 and miR482e-5p in AGL6sil and IAA9sil ovaries, respectively, conducted by stem-loop qRT-PCR. U6 rRNA was used as the internal control. Values are mean ± SEM of three biological samples. Asterisks indicate a significant difference when compared with reference ctrl sample according to Student’s t-test (*P < 0.05)

The expression of two mature miRNAs (i.e. miR393 and mir482e-5p), downregulated both in AGL6sil and IAA9sil flower buds as compared with ctrl ones, was also evaluated by an independent method. The stem-loop qRT-PCR analysis showed that miR393 and mir482e-5p expressions were reduced by approximately 54% and 37%, respectively, in AGL6sil and IAA9sil confirming the RNA-Seq results (Fig. 1).

## Discussion

IAA9 and AGL6 are transcription factors that repress ovary growth before fertilization, and their downregulation causes parthenocarpic fruit development in tomato. In this study, we have identified a set of miRNAs whose expression is modified in tomato ovary as a consequence of the downregulation of *IAA9* and *AGL6*.

In both datasets, almost all miRNAs were downregulated, suggesting that silencing of *IAA9* and *AGL6* increases the transcription of the respective miRNA targets. However, downregulation of *AGL6* caused a more pronounced effect on miRNA expression than the *IAA9* silencing in terms of number of differentially expressed miRNA. It is remarkable that five miRNAs showed altered expression in both ovary libraries suggesting an interplay between IAA9 and AGL6 signaling. Two of the identified miRNAs, miR166 and miR167, have previously been implicated in tomato parthenocarpy and fruit formation, respectively [11, 13]. Furthermore, sly-miRNA393, which is downregulated in both *IAA9*- and *AGL6*-silenced ovaries, belongs to a family, whose members control the transcription of the auxin receptor TIR1; miR393 has been shown to be repressed after fertilization in cucumber fruits [23]. In addition, miRNA482, expressed in stamen and pistil libraries and having as target the pectate lyase enzyme, has been reported to play a regulatory role during ripening in tomato [24, 25].

Further studies may clarify the role of the identified miRNAs in the initial stages of tomato fruit growth.

## Materials and methods

### Small RNA-Seq for known and novel miRNA identification

Preparation and sequencing of small RNA libraries were conducted as described [18]. Small RNA reads were mapped to the reference genome using Bowtie, and then the mapped small RNA tags were used to looking for known miRNA. miRBase20.0 was used as reference, modified software mirdeep2 [22] and srna-tools-cli were used to obtain the potential miRNA and draw the secondary structures. The characteristics of hairpin structure of miRNA precursor was used to predict novel miRNA. The available software miREvo [21] and mirdeep2 were integrated to predict novel miRNA through exploring the secondary structure. For both known and novel miRNA, custom scripts were used to obtain the miRNA counts as well as base bias on the first position of identified miRNA with certain length and on each position of all identified miRNA, respectively.

### Analysis of miRNAs expression by Stem-Loop quantitative reverse transcription PCR (qRT-PCR)

Total RNA was isolated from flower buds (*Solanum lycopersicum* UC82 cv) injected with either LDHs (ctrl) or dsRNA-LDHs (AGL6sil or IAA9sil). The stem–loop qRT-PCR primers were designed according to [26]. Stem–loop RT reactions were performed following the thermal conditions reported in [27]. Briefly, the reaction mixtures were incubated at 16 °C for 30 min, followed by 60 cycles at 30 °C for 30 s, 42 °C for 30 s, and 50 °C for 1 s. At the end, the RT enzyme was inactivated at 85 °C for 5 min. The RT reaction in a total volume of 20 µl, contained 2 µg of RNA samples, 1 µL of 1 µM stem-loop RT primer, 1 µL of 10 mM dNTP mix, 4 µL 5X First-Strand buffer, 1.3 µl of 25mM MgCl_2_, and 1 µL Improm II Reverse transcriptase (Promega). The relative expression level of target miRNAs was calculated using 2^−ΔΔCt^ method [28] normalizing against U6 (XR_003244923.1) as the reference gene. The list of primers used is reported in Additional file 5.

### Limitations

Each small RNA library was obtained from total RNA isolated from a pool of ovaries after treatment with exogenous dsRNA. The detection of differentially expressed miRNAs was performed by comparing single IAA9sil, AGL6sil, and crtl libraries. Data could be strengthened by performing the same analysis on a different tomato cultivar.

### Electronic supplementary material

Below is the link to the electronic supplementary material.

Supplementary Material 1

Supplementary Material 2

Supplementary Material 3

Supplementary Material 4

Supplementary Material 5

## Data Availability

The data discussed in the publication have been deposited in NCBI’s Gene Expression Omnibus [29] and are accessible through GEO Series accession number GSE225319 (https://www.ncbi.nlm.nih.gov/geo/query/acc.cgi?acc=GSE225319).
