# Supplementary material for: MicroRNAs associated with AGL6 and IAA9 function in tomato fruit set
Source: BMC Res Notes. 2023 Sep 30;16:242. doi: 10.1186/s13104-023-06510-z (PMC10544166; doi:10.1186/s13104-023-06510-z)
Supplement: Supplementary file 2 — Supplementary Material 2 [file 13104_2023_6510_MOESM2_ESM.docx]

# **
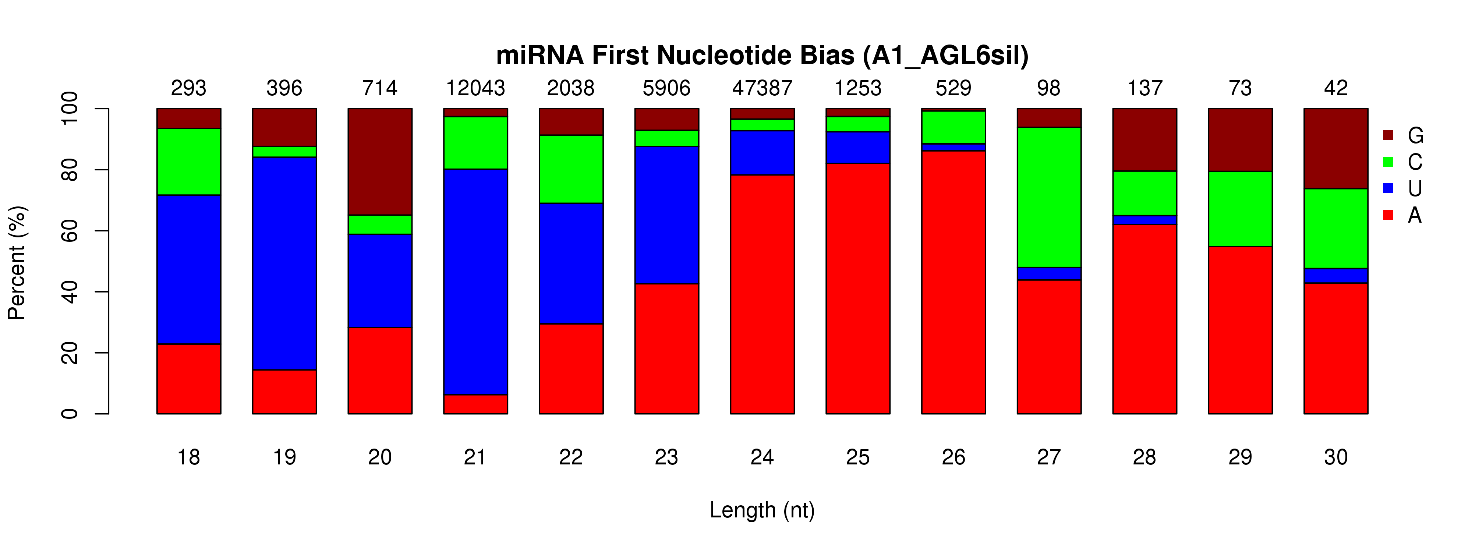

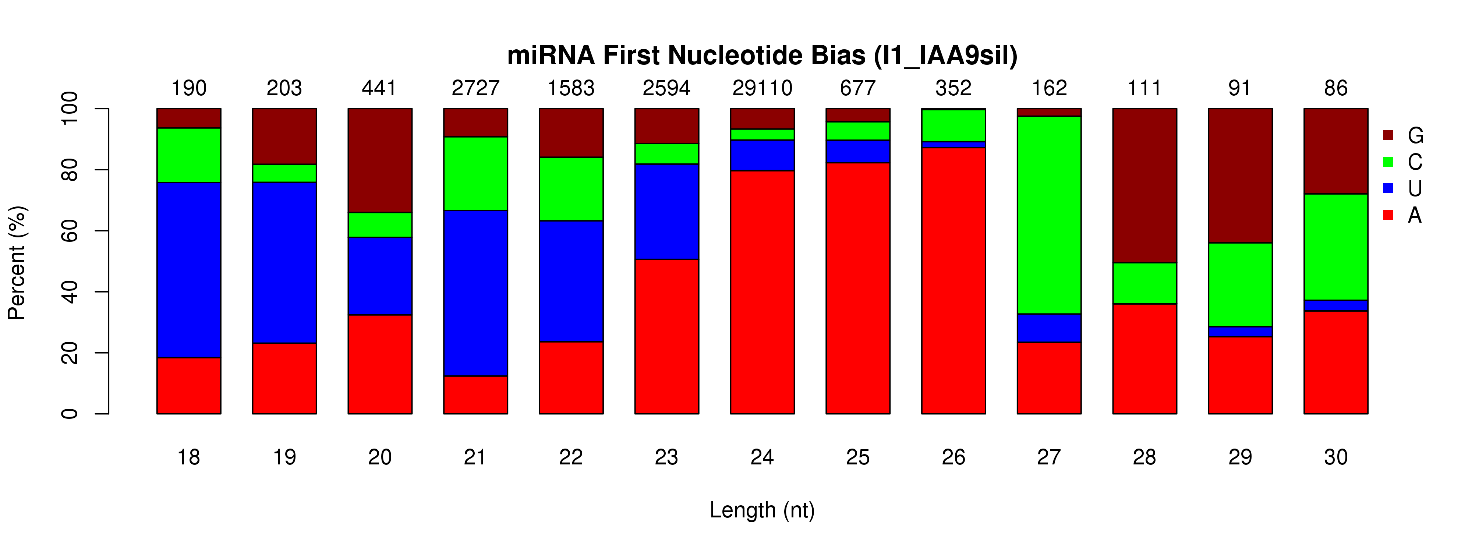

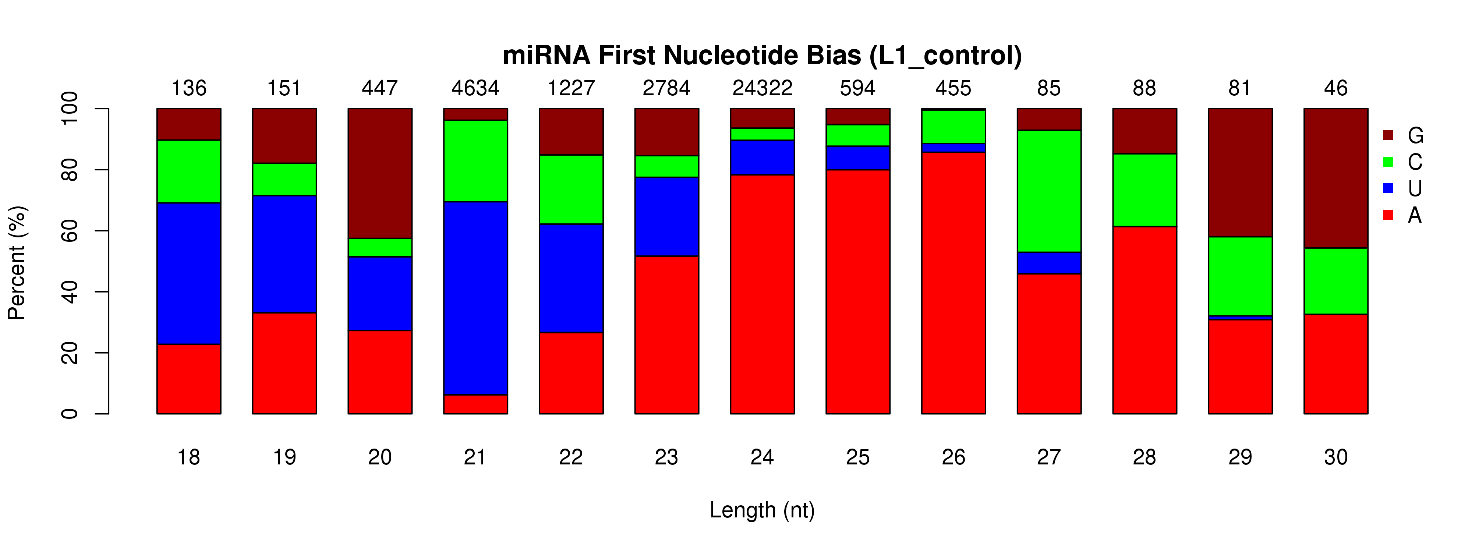
**

# **Additional file 2**. First base preference of novel miRNAs of 18 to 30 nucleotides (nt) in length. The horizontal axis is the length of the miRNA and the vertical axis is the ratio of A/U/C/G in the first base of the miRNA according to the miRNA length. The numbers on the columns are the total numbers of miRNAs with specific length.
