# Supplementary material for: MicroRNAs associated with AGL6 and IAA9 function in tomato fruit set
Source: BMC Res Notes. 2023 Sep 30;16:242. doi: 10.1186/s13104-023-06510-z (PMC10544166; doi:10.1186/s13104-023-06510-z)
Supplement: Supplementary file 4 — Supplementary Material 4 [file 13104_2023_6510_MOESM4_ESM.docx]

**Additional file 4. Novel differentially expressed miRNAs**

| **miRNAs** | **Fold change**  **(IAA9sil vs ctrl)** | **Mature sequence** |
| --- | --- | --- |
| novel_220 | -1.76 | guucccuugaucacuucaug |
| novel_4 | -1.64 | uugaaguguuugagggaacuc |
| novel_81 | -2.50 | gcugaaguguuuaggggaacu |
| novel_93 | 2.23 | aaucuuggaauguuuugcuuuggu |
| novel_521 | 1.91 | acauggugcucaucugacaguuga |
| novel_110 | 1.55 | aagauucguuauguauacaugaga |
| novel_122 | -1.70 | auaacggcuacggauuuuccuugu |
| novel_124 | -1.58 | cuuccgaucaggacaaguggguug |
| novel_25 | -1.52 | aacgaucuucgacuagcaaaagac |
| novel_225 | -1.76 | ggucaugcucggacagccucacu |
| novel_72 | -2.73 | auauucggucuuauuacauugaug |
| novel_318 | 1.81 | uguauuauauuguaggacacgugu |
| novel_79 | 1.52 | gaauggauaagauuaugacacgug |
| novel_360 | 1.88 | aucugagauaucgaauacacgagc |
| novel_522 | -1.52 | acaaauggauaagauggagaggug |
| novel_425 | -2.12 | aagacuaaagagacuucacgagga |
| novel_170 | 2.09 | auucggugucgaguagauguaugu |
| novel_363 | -2.43 | acucgaucaacgcuggcuacuggu |
| **miRNAs** | **Fold change**  **(AGL6sil vs ctrl)** | **Mature sequence** |
| novel_264 | -3.13 | gcagcaccauuaagauucac |
| novel_296 | -3.58 | gauuugagcuuggaacuucca |
| novel_351 | 2.01 | ugaagguccgagguugagguu |
| novel_551 | -2.32 | aacaaguccaacaagaaaaacgug |
| novel_108 | 1.89 | auugugggauuucauugaauauga |
| novel_316 | 1.94 | aauuuccggucugguucaucc |
| novel_327 | -2.09 | aaggagugugccacauaagacaaa |
| novel_69 | 2.38 | uucccuugaccgcuucauuag |
| novel_146 | 1.63 | aaaucugauuggucgaauggaguc |
| novel_493 | 1.54 | uauuauguuauguagaaggagugu |
| novel_267 | 7.94 | aauugucauguaggacgaacgugu |
| novel_304 | -3.03 | ucgggugcuugucugucuuaa |
| novel_516 | -2.75 | acacuucuguugaacaaagcgaga |
| novel_281 | -3.36 | agguugauguacguuacuguaagu |
| novel_336 | -2.77 | uugugacguguaggguucaucugc |
| novel_352 | 1.83 | ugacuaccugaacuuucacuuugu |
| novel_326 | -1.58 | ccuccauggcugggagcugccu |
| novel_25 | -1.55 | aacgaucuucgacuagcaaaagac |
| novel_36 | -1.69 | agacugugacuuauuugaaaagac |
| novel_225 | -4.35 | ggucaugcucggacagccucacu |
| novel_60 | 1.83 | aaagauccucggacuuucaggcgg |
| novel_252 | -1.80 | aaucaaaaucgcugcugaccuagc |
| novel_77 | 1.91 | uugugguguauuagugagacugau |
| novel_476 | 2.04 | augucguguguccuaucaauugaa |
| novel_104 | 2.50 | aacggauuagaucggauuagauuu |
| novel_522 | -3.20 | acaaauggauaagauggagaggug |
| novel_343 | 1.63 | aacgaucucuggauaacuuagcgg |
| novel_307 | 3.64 | auuaaguggcucuuucguacgaca |
| novel_127 | 2.08 | uuagucucuagaaauucuacuugg |
| novel_242 | -1.95 | uaucuuguugucgcggcuacag |
| novel_143 | 1.63 | uuauaacauagugaugggacgagu |
| novel_441 | -1.74 | aauucagauugugaccuaccgcuc |
| novel_170 | 3.00 | auucggugucgaguagauguaugu |
