# Supplementary material for: MicroRNAs associated with AGL6 and IAA9 function in tomato fruit set
Source: BMC Res Notes. 2023 Sep 30;16:242. doi: 10.1186/s13104-023-06510-z (PMC10544166; doi:10.1186/s13104-023-06510-z)
Supplement: Supplementary file 5 — Supplementary Material 5 [file 13104_2023_6510_MOESM5_ESM.docx]

| Target | Primer | Sequence (5’→ 3’) |
| --- | --- | --- |
| miR393 | Stem loop RT | GTCGTATCCAGTGCAGGGTCCGAGGTATTCGCACTGGATACGACATTCCG |
|  | Forward | GCCAGATCATGCGATCTCTT |
| miR482e-5p | Stem loop RT | GTCGTATCCAGTGCAGGGTCCGAGGTATTCGCACTGGATACGACAATCTT |
|  | Forward | TCTAATGTGGGTGGGGTGGA |
| U6 | RT | TATCCAGTGCAGGGTCCGAGGTATTTTGTGCGTGTCATCCTTGCG |
|  | Forward | GGAACGATACAGAGAAGATTAGCA |
| Universal | Reverse | GTGCAGGGTCCGAGGT |

**Additional file 5. List of primers**
